# Ultrasound-Guided 5% Dextrose Hydrodissection Procedures for Persistent and Recurrent Post-Surgical Carpal Tunnel Syndrome: A Prospective Single-Center Cohort Study

**DOI:** 10.3390/diagnostics16132106

**Published:** 2026-07-05

**Authors:** Marius Nicolae Popescu, Claudiu Căpeț, Simona Elena Săvulescu, Cristina Popescu, Mihai Berteanu

**Affiliations:** 1 Department of Physical and Rehabilitation Medicine, Elias Emergency University Hospital, Carol Davila University of Medicine and Pharmacy, 020021 Bucharest, Romania; marius.popescu@umfcd.ro (M.N.P.); claudiu.capet@gmail.com (C.C.); mberteanu@gmail.com (M.B.); 2 Clinic of Physical and Rehabilitation Medicine, Elias Emergency University Hospital, 011461 Bucharest, Romania; 3 Department of Oncologic Dermatology, Elias Emergency University Hospital, Carol Davila University of Medicine and Pharmacy, 020021 Bucharest, Romania

**Keywords:** carpal tunnel syndrome, recurrent carpal tunnel syndrome, persistent carpal tunnel syndrome, hydrodissection, 5% dextrose, ultrasound-guided injection, post-surgical neuropathy

## Abstract

**Background/Objectives**: Persistent and recurrent symptoms after carpal tunnel release remain challenging, and non-surgical options for post-surgical carpal tunnel syndrome (CTS) are poorly defined. This study evaluated 6-month treatment success after ultrasound-guided 5% dextrose in water (D5W) hydrodissection in persistent or recurrent post-surgical CTS and compared outcomes between subgroups. **Methods**: In this prospective single-center interventional cohort study, 100 patients with post-surgical CTS were enrolled: 50 with persistent disease and 50 with recurrent disease. All underwent 1–4 treatments with ultrasound-guided D5W hydrodissection using a standardized protocol. Treatment success at 6 months was defined as Patient Global Impression of Change (PGIC) scores of 6–7 together with clinically meaningful improvement in at least two of three domains: ≥30% reduction in 0–10 VAS pain, ≥0.8-point reduction in BCTQ-SSS, and ≥0.5-point reduction in BCTQ-FSS. Secondary outcomes included 12-month durability; longitudinal clinical, ultrasonographic, and electrodiagnostic changes; predictors of treatment success; treatment exposure; and safety. **Results**: Treatment success at 6 months occurred in 58 of 100 patients (58.0%) and was more frequent in recurrent than persistent CTS (70.0% vs. 46.0%, *p* = 0.015). At 12 months, treatment success persisted in 52.0% of the cohort (64.0% vs. 40.0%, *p* = 0.017). Clinical and ultrasonographic outcomes improved significantly over time, with greater improvement in recurrent CTS. Electrodiagnostic improvement was more modest. Recurrent CTS independently predicted success (adjusted OR 2.58, 95% CI 1.10–6.03), whereas severe electrodiagnostic involvement and greater baseline median nerve cross-sectional area were associated with lower odds of success. No serious treatment-related adverse events occurred. **Conclusions**: Ultrasound-guided D5W hydrodissection was associated with meaningful improvement in post-surgical CTS, particularly recurrent disease.

## 1. Introduction

Carpal tunnel syndrome (CTS) is the most common entrapment neuropathy of the upper limb, and surgical decompression is generally effective. A minority of patients continue to report persistent, recurrent, or new symptoms after surgery, creating a clinically challenging postoperative group in whom further management is often less straightforward than in primary disease [1,2]. Recent reviews of failed carpal tunnel decompression continue to emphasize that postoperative symptoms require structured reassessment rather than automatic progression to revision surgery, particularly because the underlying causes may differ substantially across patients [3,4].

This distinction is especially important for persistent and recurrent CTS. Persistent symptoms after surgery are often linked to incomplete release, incorrect or incomplete diagnosis, severe pre-existing neuropathy, or proximal contributors [2,3], whereas recurrent symptoms are more often associated with perineural fibrosis, scar-related tethering, or renewed compression after an initial period of improvement [2]. Although revision surgery can improve outcomes in selected patients, it remains a second-line strategy with less predictable results than primary release and with a more complex diagnostic and technical context. A clinically useful non-surgical option in this setting would address a real and unmet need [2,5].

Ultrasound-guided hydrodissection with 5% dextrose in water (D5W) has emerged as a plausible treatment for CTS because it combines a mechanical effect—separation of the median nerve from surrounding tissues—with a potentially favorable injectate profile. In primary CTS, randomized and comparative studies have shown clinically meaningful benefit of perineural D5W injection [6,7], and recent systematic reviews and network meta-analyses have placed dextrose among the more promising injectates for symptom and functional improvement [8,9]. However, evidence in post-surgical CTS remains sparse. To date, the most directly relevant study is the retrospective cohort reported by Chao et al., which suggested clinically important and durable benefit of D5W hydrodissection in patients with persistent or recurrent symptoms after prior release [10]. Prospective data in this specific population remain limited.

The post-surgical setting also requires more than symptom reporting alone. In patients with persistent or recurrent symptoms after surgery, clinical outcomes need to be interpreted together with structural and neurophysiologic information. Recent literature on failed decompression highlights the role of nerve conduction studies and imaging in clarifying the mechanism of failure and supporting further decision-making [4,11]. Ultrasound is particularly attractive in this context because it may help identify postoperative structural abnormalities while also providing a noninvasive means of quantifying median nerve morphology over time [11,12]. A study design combining clinical, ultrasonographic, and electrodiagnostic assessment may therefore provide a more clinically useful picture of response than any single domain alone.

To our knowledge, no prospective study has examined D5W hydrodissection specifically in post-surgical CTS, and no prior work has prospectively compared persistent and recurrent disease as a prespecified objective. The only directly relevant series—Chao et al.—was retrospective and relied on a single patient-reported outcome, without ultrasonographic or electrodiagnostic follow-up. The primary aim was to evaluate 6-month treatment success after ultrasound-guided 5% dextrose hydrodissection in patients with persistent or recurrent post-surgical CTS. Secondary aims were to compare persistent and recurrent subgroups; characterize longitudinal clinical, ultrasonographic, and electrodiagnostic changes; assess durability of response at 12 months; explore baseline predictors of treatment success; and describe treatment exposure and safety. We hypothesized that outcomes would be more favorable in recurrent CTS than in persistent CTS.

## 2. Materials and Methods

### 2.1. Study Design

This was a prospective single-center uncontrolled cohort study designed to evaluate clinical, ultrasonographic, and electrodiagnostic outcomes after ultrasound-guided 5% dextrose hydrodissection in patients with persistent or recurrent symptoms after prior carpal tunnel release. The study was conducted and reported in accordance with the Strengthening the Reporting of Observational Studies in Epidemiology (STROBE) recommendations for cohort studies [13].

### 2.2. Setting and Recruitment

The study was conducted at the Elias University Emergency Hospital, Bucharest, Romania. Recruitment took place between March 2024 and February 2025. Follow-up continued after completion of recruitment, and the final 12-month assessments were completed after the last enrolled participants reached the 12-month follow-up visit. As a comparison between persistent and recurrent post-surgical CTS was prespecified, recruitment was planned to achieve approximately balanced subgroup sizes.

### 2.3. Participants and Subgroup Classification

Adults with persistent or recurrent symptoms after previous open or endoscopic carpal tunnel release were eligible if they had current symptoms compatible with CTS, electrodiagnostic evidence of median neuropathy at the wrist, and ultrasonographic evidence of median nerve enlargement at the carpal tunnel inlet, defined as a median nerve cross-sectional area > 10 mm^2^ [14]. Only patients considered suitable for a non-surgical treatment approach were included, and all participants provided written informed consent.

Persistent CTS was defined as symptoms compatible with CTS persisting for more than 3 months after surgery, without a symptom-free interval and without clinically satisfactory postoperative relief. Recurrent CTS was defined as recurrence of CTS symptoms after an initial period of clinically satisfactory postoperative improvement, with a symptom-free interval of at least 6 months [2,15]. In both subgroups, classification required concordance between clinical, electrodiagnostic, and ultrasonographic findings.

Patients were excluded if they had progressive motor deficit, strong suspicion of incomplete release requiring prompt surgical reassessment, recent corticosteroid injection, pregnancy or breastfeeding, known dextrose intolerance, coagulation-related contraindications to injection, or an alternative diagnosis considered more likely to explain symptoms on the basis of clinical examination, electrodiagnostic testing, and/or ultrasound assessment. Such diagnoses included proximal median neuropathy, cervical radiculopathy, generalized polyneuropathy, inflammatory wrist arthropathy, or another disorder judged to better explain the patient’s symptoms. Patients with known hereditary neuropathy with liability to pressure palsies, known amyloidosis, or other systemic or generalized neuropathic disorders associated with recurrent or multifocal entrapment neuropathies were also excluded.

Patients were considered eligible for ultrasound-guided hydrodissection when symptoms remained clinically relevant, objective findings supported ongoing median neuropathy at the wrist, and no immediate indication for revision surgery was present.

### 2.4. Intervention

All patients underwent ultrasound-guided hydrodissection with 5% dextrose in water according to a standardized procedural protocol. D5W was selected as the injectate based on prior CTS literature suggesting clinically meaningful benefits and a favorable safety profile, including in comparison with corticosteroid injection, while providing a non-corticosteroid option for a post-surgical population [8,10,16]. All procedures were performed under real-time ultrasound guidance using an in-plane radial-to-ulnar approach at the proximal carpal tunnel. Before needle advancement, the median nerve, flexor tendons, ulnar artery, and adjacent superficial neural and vascular structures were identified sonographically. This trajectory was selected as the standardized operator approach because, in our practice, the ulnar artery was frequently superficial or close to the expected ulnar entry path, making a strictly ulnar-sided entry less favorable. The needle tip was advanced under continuous visualization into the perineural plane, with care taken to avoid the palmar cutaneous branch region and adjacent vascular structures. The target was the perineural space around the median nerve, with the aim of achieving circumferential hydrodissection and separation of the median nerve from the transverse carpal ligament superficially and from the adjacent flexor tendons and surrounding soft tissues deeply. A total volume of 10 mL of 5% dextrose in water was injected at each session [17]. All procedures were performed by a single physician with extensive experience in ultrasound-guided perineural injections [18,19,20]. A high-frequency linear transducer (GE HealthCare Venue Go, 12L-RS linear probe, 3.4–12.6 MHz) and a 21-gauge needle were used. No local anesthetic was injected into the perineural space. Hydrodissection was considered technically adequate when circumferential fluid separation around the median nerve was achieved under real-time ultrasound guidance. The procedure followed the same structured ultrasound-guided approach to upper-limb anatomy that we previously used in the EUH series on ultrasound-guided interventions in the upper limb [21,22].

Additional injections could be administered according to the prespecified treatment algorithm if response remained incomplete at follow-up reassessment.

### 2.5. Treatment Algorithm

All patients received one baseline ultrasound-guided 5% dextrose hydrodissection procedure at week 0. Follow-up reassessments were scheduled at weeks 4, 8, and 12. Repeat injection was considered only when patients had ongoing CTS-compatible symptoms that remained clinically relevant to the patient, such as persistent pain, paresthesia, nocturnal symptoms, or functional limitation attributable to median neuropathy at the wrist, with no contraindication to further injection and no new indication for revision surgery. Retreatment was allowed if at least one prespecified incomplete-response criterion was present: (1) VAS reduction <50% from baseline, (2) BCTQ-SSS reduction <0.8 points from baseline, or (3) absence of satisfactory patient-reported global improvement. No further injections were given once treatment success criteria had been met or when additional injection was judged unlikely to provide meaningful benefit. The maximum number of injections was four.

### 2.6. Concomitant Treatments

Use of stable oral analgesic medication was permitted during follow-up if unchanged from baseline. Night splinting and previously established home exercise routines were allowed if maintained without escalation. No corticosteroid injection, non-study perineural injection, or other injection treatment targeting the study wrist was permitted before completion of the 6-month primary endpoint assessment. Repeat D5W hydrodissection procedures performed according to the prespecified study algorithm were allowed. Any co-intervention considered likely to materially influence symptom status was recorded and reviewed as a potential protocol deviation, including newly initiated structured rehabilitation program targeting the study wrist, changes in splint use, escalation of analgesic treatment, or return to heavy repetitive manual activity during follow-up.

### 2.7. Outcome Assessment

Clinical outcomes included pain intensity assessed by the visual analogue scale (VAS); symptom severity and functional status were assessed by the Boston Carpal Tunnel Questionnaire Symptom Severity Scale (BCTQ-SSS) and Boston Functional Status Scale (BCTQ-FSS) [23], and patient global improvement was assessed by the Patient Global Impression of Change (PGIC) [24,25]. Ultrasonographic outcomes included the median nerve cross-sectional area at the carpal tunnel inlet [2]. Electrodiagnostic assessment included sensory and motor conduction parameters and severity classification based on the Bland system.

Assessments were performed at baseline and during follow-up according to the study schedule. Clinical outcomes were collected at all planned visits. Ultrasound assessments were obtained at all planned visits. Electrodiagnostic reassessment was scheduled at 6 and 12 months rather than at the 3-month visit, consistent with evidence that neurophysiologic recovery in compressive neuropathy lags clinical improvement and that meaningful electrophysiologic change is unlikely to be detectable within 3 months of intervention [26,27]; this approach also minimized the burden associated with serial electrodiagnostic testing.

### 2.8. Assessment Standardization

Clinical, ultrasonographic, and electrodiagnostic assessments were performed according to predefined study procedures. Ultrasound measurements were obtained at fixed anatomical landmarks, with median nerve cross-sectional area measured at the carpal tunnel inlet in a transverse plane, at the level of the pisiform [28]. Probe orientation and image acquisition plane were kept consistent across visits, with care taken to minimize transducer pressure. Electrodiagnostic severity was classified according to the Bland neurophysiological grading scale for carpal tunnel syndrome [29,30] and collapsed into three categories for analysis: mild (grades 1–2), moderate (grades 3–4), and severe (grades 5–6). Electrodiagnostic assessment included median sensory and motor nerve conduction studies at the wrist; needle electromyography was performed when clinically indicated to exclude alternative or more proximal neuropathic processes. Clinical outcome assessors were not blinded to subgroup classification, which may have introduced performance and detection bias in between-group comparisons.

### 2.9. Primary Endpoint

Six months was selected as the primary endpoint to capture treatment efficacy after completion of the injection algorithm, whereas 12-month assessment was included to evaluate durability of response. Treatment success at 6 months was defined as a PGIC rating of 6 or 7 (“much improved” or “very much improved”) together with clinically meaningful improvement in at least 2 of the following 3 domains: VAS pain reduction of at least 30% from baseline, BCTQ-SSS reduction of at least 0.8 points, and BCTQ-FSS reduction of at least 0.5 points. Because no established composite responder definition has been validated specifically for post-surgical CTS treated with D5W hydrodissection, treatment success was defined before the primary analysis as a conservative multidomain outcome combining patient-perceived global improvement with clinically meaningful changes in pain, symptom severity, and functional status. The same criteria were applied at 12 months for the assessment of maintained treatment success. Patients with missing outcome data at the corresponding time point and those undergoing revision surgery before that assessment were classified as non-successes in the full analysis set.

The threshold of 0.8 points for BCTQ-SSS was selected to exceed the minimally important clinical difference reported in the literature and to ensure that only patients with clinically robust symptom improvement were classified as successes [31,32,33].

PGIC was coded so that higher scores indicated greater improvement. Clinically meaningful global improvement was defined as a PGIC rating of 6 (“much improved”) or 7 (“very much improved”).

### 2.10. Secondary Outcome Measures

Secondary outcome measures included longitudinal clinical change, longitudinal ultrasonographic and electrodiagnostic change, treatment success at 12 months, durability of response among 6-month responders, number of injections administered and safety outcomes including adverse events and revision surgery.

### 2.11. Statistical Analysis

All analyses were performed using R (version 4.2.0). All tests were two-sided, and *p* < 0.05 was considered statistically significant for the primary analysis. Continuous variables were summarized as the mean ± SD or median (IQR), as appropriate, and categorical variables were summarized as counts and percentages.

The unit of analysis was the individual patient, with only one hand included per participant. Baseline characteristics were summarized for the overall cohort and by CTS subtype (persistent vs. recurrent). Between-group comparisons used Student’s *t*-test or Mann–Whitney U test for continuous variables and chi-square or Fisher’s exact test for categorical variables, as appropriate.

The primary endpoint was treatment success at 6 months according to the prespecified composite definition. Success rates were reported with 95% confidence intervals for the overall cohort and by CTS subtype, and between-group differences were described using odds ratios with 95% confidence intervals.

Longitudinal changes in VAS, BCTQ-SSS, BCTQ-FSS, and ultrasonographic parameters were analyzed using linear mixed-effects models with patient-level random intercepts and fixed effects for time, CTS subtype, and the time-by-subtype interaction [34,35].

Baseline predictors of 6-month treatment success were first examined in univariable logistic regression analyses. Baseline BCTQ-FSS was not included in the predictor analysis to avoid multiplying closely related severity measures, given that BCTQ-SSS was already included as the symptom-specific predictor and functional status was captured as a component of the composite primary endpoint. Baseline VAS was similarly excluded due to collinearity with BCTQ-SSS; as the more CTS-specific symptom measure, BCTQ-SSS was retained as the sole symptom severity predictor in the regression analyses. A parsimonious multivariable logistic regression model was then constructed using clinically prioritized baseline variables, with model complexity restricted according to the number of observed outcome events. Adjusted odds ratios with 95% confidence intervals were reported, and model performance was assessed by discrimination, calibration, and bootstrap internal validation.

For repeated continuous outcomes, mixed-effects models used all available data under a missing-at-random assumption [35,36]. For the primary binary endpoint, missing outcome data and revision surgery before the corresponding assessment were conservatively classified as non-successes in the full analysis set. Safety analyses included all patients receiving at least one injection. The per-protocol population comprised all enrolled patients who completed the 6-month primary endpoint assessment without major protocol deviations, without newly initiated co-interventions judged likely to materially influence symptom status, and without revision surgery before that assessment. A sensitivity analysis of the primary endpoint was performed in this population.

A formal a priori sample size calculation was not feasible because prospective effect-size data in this specific population were unavailable at the time of study design. Recruitment was therefore planned pragmatically, targeting approximately 50 patients per subgroup based on the only directly relevant retrospective study [10]. Accordingly, effect estimates are presented with 95% confidence intervals, and the study should be interpreted as prospective hypothesis-generating evidence rather than definitive confirmatory evidence.

### 2.12. Ethics

The study was conducted in accordance with the Declaration of Helsinki and approved by the local institutional ethics committee. All participants provided written informed consent before enrollment.

## 3. Results

### 3.1. Participant Flow

**Figure 1:**
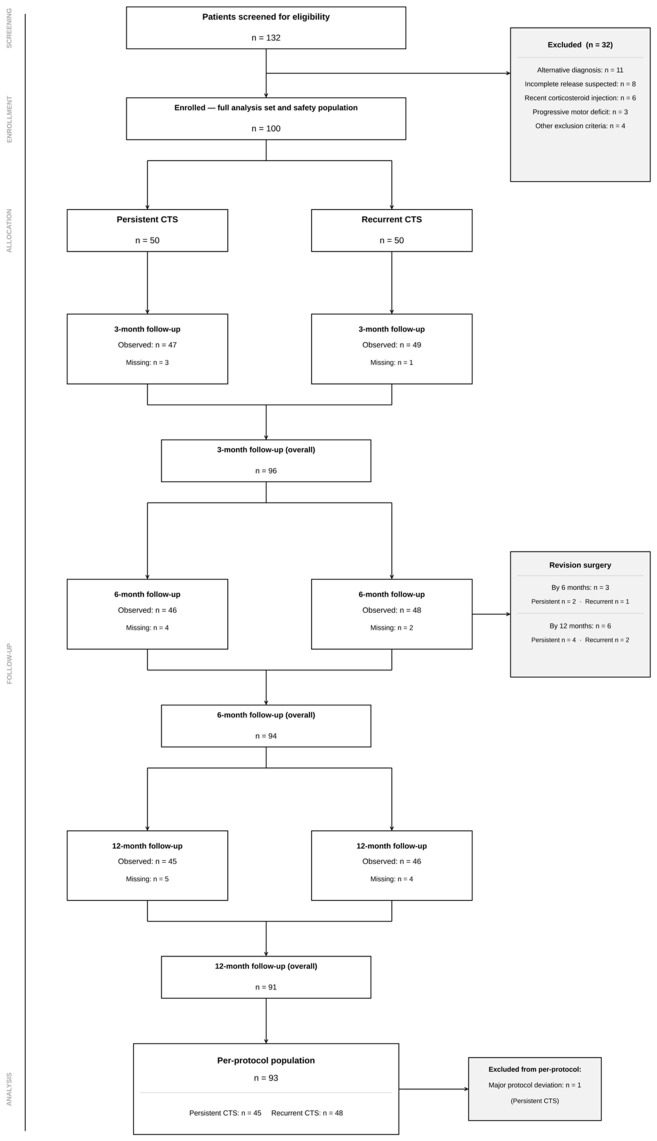
Study flow diagram. Screening, enrollment, follow-up, and analysis populations.

Between March 2024 and February 2025, 132 patients with persistent or recurrent symptoms after prior carpal tunnel release were screened. Thirty-two screened patients were excluded for prespecified reasons (Figure 1), including 11 with an alternative diagnosis judged more likely, 8 with suspected incomplete release requiring surgical reassessment, 6 with recent corticosteroid injection, 3 with progressive motor deficits, and 4 with other prespecified exclusion criteria, leaving 100 patients who underwent at least one ultrasound-guided hydrodissection with 5% dextrose: 50 with persistent CTS and 50 with recurrent CTS. All were included in the full analysis and safety populations. Follow-up data were available for 96 patients at 3 months, 94 at 6 months, and 91 at 12 months. Three patients underwent revision surgery before the 6-month assessment and were classified as non-successes in the primary analysis. Cumulative revision surgery reached 6 cases by 12 months. The per-protocol population comprised 93 patients. One patient who completed the 6-month primary endpoint assessment was excluded from the per-protocol population because of a major protocol deviation consisting of a newly initiated structured rehabilitation program targeting the study wrist before the primary endpoint assessment (Figure 1).

### 3.2. Baseline Characteristics

Baseline characteristics are shown in Table 1. Mean age was 56.2 ± 11.7 years. 71.0% of patients were women, and 28.0% had diabetes mellitus. Open carpal tunnel release had been performed in 72.0% of cases.

Compared with the recurrent subgroup, patients with persistent CTS presented with a heavier clinical burden at baseline: they were older and had higher baseline pain intensity, while time since surgery, duration of current symptoms, diabetes prevalence, electrodiagnostic severity, and BCTQ-SSS tended to be less favorable numerically. Although the overall distribution of electrodiagnostic severity categories did not differ significantly between subgroups (*p* = 0.29), severe involvement was numerically more frequent in persistent than in recurrent CTS (40.0% vs. 24.0%). These differences limit direct between-group comparisons and are acknowledged in the adjusted analyses.

### 3.3. Treatment Exposure

The median number of injections administered was 2 (IQR 1–3) overall, 3 (IQR 2–3) in persistent CTS, and 2 (IQR 1–2) in recurrent CTS (*p* = 0.003). One, two, three, and four injections were administered in 31, 29, 24, and 16 patients, respectively. Recurrent CTS more often reached satisfactory improvement after one or two injections, whereas persistent CTS more frequently required repeated treatment.

### 3.4. Protocol Deviations and Co-Interventions

One major co-intervention judged likely to materially influence symptom status was documented before completion of the 6-month primary endpoint assessment, consisting of a newly initiated structured rehabilitation program targeting the study wrist in one patient. This patient remained in the full analysis set but was excluded from the per-protocol analysis.

### 3.5. Primary Outcome

At 6 months, treatment success in the full analysis set was observed in 58 of 100 patients (58.0%). Success was more frequent in recurrent CTS than in persistent CTS (70.0% vs. 46.0%, *p* = 0.015) (Table 2). A sensitivity analysis restricted to the per-protocol population (n = 93) yielded consistent results: treatment success was observed in 58 of 93 patients (62.4%), with subgroup differences in similar direction and magnitude (persistent CTS: 51.1%; recurrent CTS: 72.9%), supporting the robustness of the primary analysis findings.

Because the two subgroups differed in baseline clinical burden, an adjusted analysis was also performed using the final parsimonious multivariable model. Recurrent CTS remained independently associated with higher odds of 6-month treatment success than persistent CTS (adjusted OR 2.63, 95% CI 1.14–6.10; *p* = 0.024).

### 3.6. Secondary Outcomes

Secondary outcomes at 6 and 12 months are summarized in Table 2. At 12 months, treatment success was still observed in 52.0% of the full cohort and remained more frequent in recurrent CTS than in persistent CTS (64.0% vs. 40.0%, *p* = 0.016). Among 6-month responders, 87.9% maintained response at 12 months, without a significant subgroup difference. PGIC-defined marked improvement and a CSA reduction of at least 2 mm^2^ showed the same overall pattern, with higher rates in recurrent CTS at both follow-up points. By contrast, improvement by at least one Bland category was less frequent and did not differ significantly between subgroups. Revision surgery was uncommon, reaching 3.0% by 6 months and 6.0% by 12 months. Absolute between-group differences with corresponding 95% confidence intervals are reported in Table 2 for the principal binary outcomes.

### 3.7. Longitudinal Clinical and Ultrasonographic Outcomes

**Figure 2:**
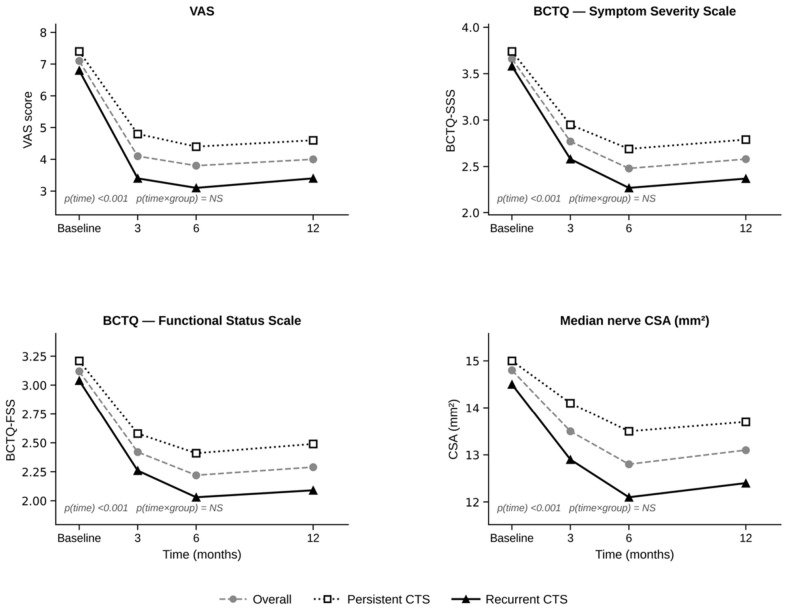
Longitudinal changes in clinical and ultrasonographic outcomes. Observed mean values for VAS, BCTQ-SSS, BCTQ-FSS, and median nerve cross-sectional area over follow-up, overall and by subgroup.

Longitudinal changes in clinical and ultrasonographic parameters are presented in Table 3 and Figure 2. Mean VAS declined from 7.1 ± 1.3 at baseline to 4.1 ± 2.1 at 3 months, 3.7 ± 1.4 at 6 months, and 3.8 ± 1.3 at 12 months. BCTQ-SSS and BCTQ-FSS followed a similar course, with the largest improvements observed by 3 to 6 months and broad maintenance thereafter. In mixed-effects models, VAS, BCTQ-SSS, BCTQ-FSS, and CSA improved significantly over time (all *p* < 0.001). However, time-by-group interactions were not statistically significant, indicating that the longitudinal trajectories did not differ clearly between persistent and recurrent CTS. Ultrasonographic findings changed in the same direction. Mean CSA decreased from 14.7 ± 2.7 mm^2^ at baseline to 12.6 ± 3.1 mm^2^ at 6 months, with relative stability at 12 months.

### 3.8. Electrodiagnostic Outcomes

Electrodiagnostic improvement was more modest than clinical and ultrasonographic improvement. Improvement by at least one Bland severity category was observed in 27 of 94 evaluable patients (28.7%) at 6 months and in 35 of 91 (38.5%) at 12 months. Sensory and motor conduction parameters showed modest improvement over follow-up, with numerically greater changes in recurrent CTS, although these changes were smaller in magnitude than the corresponding clinical and ultrasonographic changes (Supplementary Table S1).

### 3.9. Predictors of 6-Month Treatment Success

**Figure 3:**
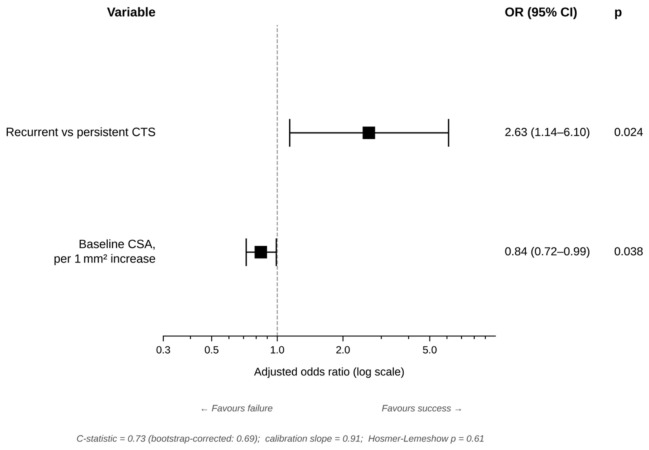
Multivariable predictors of 6-month treatment success. Forest plot showing adjusted odds ratios and 95% confidence intervals from the final parsimonious multivariable logistic regression model.

Univariable analyses are shown in Table 4. Recurrent CTS was associated with higher odds of treatment success at 6 months (odds ratio [OR] 2.74, 95% confidence interval [CI] 1.20–6.23; *p* = 0.016), whereas greater baseline median nerve CSA and longer time since surgery were associated with lower odds of success. Severe electrodiagnostic abnormality showed a directionally unfavorable association that did not reach conventional statistical significance, and diabetes, baseline symptom severity, baseline pain intensity, and symptom duration showed weaker or nonsignificant associations. In the final parsimonious multivariable model (Table 5, Figure 3), recurrent CTS remained independently associated with higher odds of treatment success (adjusted OR 2.63, 95% CI 1.14–6.10; *p* = 0.024), whereas greater baseline median nerve CSA was independently associated with lower odds of success (adjusted OR per 1 mm^2^ increase 0.84, 95% CI 0.72–0.99; *p* = 0.038). Model performance was acceptable and is summarized in Table 5.

### 3.10. Safety

Safety outcomes are presented in Table 6. Twenty-two patients (22.0%) experienced at least one adverse event. All were minor or moderate. The most common events were injection-site pain lasting more than 24 h, transient paresthesia, and bruising/ecchymosis. All episodes of transient paresthesia were self-limited and resolved without persistent neurological deficit. Hematoma requiring observation occurred in 2.0% of patients, and prolonged pain lasting more than 48 h occurred in 1.0%. No infection, tendon injury, nerve injury, or serious treatment-related adverse event was observed.

## 4. Discussion

This prospective single-center cohort study evaluated ultrasound-guided 5% dextrose hydrodissection in patients with persistent or recurrent symptoms after prior carpal tunnel release. At 6 months, treatment success was observed in 58.0% of the cohort, with consistently better outcomes in recurrent than in persistent CTS. Greater baseline median nerve enlargement was associated with a lower likelihood of treatment success, and no serious treatment-related adverse events were observed.

Post-surgical CTS remains comparatively understudied relative to primary disease, and patients with persistent or recurrent postoperative symptoms occupy a heterogeneous and clinically demanding category between further diagnostic reassessment, symptomatic management, and revision surgery [10]. Persistent and recurrent post-surgical CTS are not equivalent clinical entities: persistent symptoms more often raise concern for incomplete release, diagnostic error, proximal nerve pathology, or advanced pre-existing neural injury, whereas recurrent symptoms are more commonly linked to scar formation, renewed tethering, or recurrent compression after an initial symptom-free interval [2,4]. This distinction is important when interpreting the outcomes, because hydrodissection is more likely to work when scar-related adherence and impaired neural gliding are major contributors and less likely to help when symptoms reflect structural failure of the original decompression or an alternative diagnosis.

A 6-month treatment success rate of 58.0% compares reasonably with the effective outcome rate reported by Chao et al. in the only directly comparable post-surgical D5W series [10], despite the stricter composite endpoint used in the present study, which required concurrent improvement in pain, symptom severity, function, and patient global impression. This approach was intentionally conservative and likely provides a more clinically robust estimate of benefit than reliance on a single symptom threshold alone.

Although post-surgical data remain limited, the broader CTS literature supports the rationale for D5W hydrodissection. Randomized and comparative studies in primary CTS have shown beneficial effects of D5W injection, and recent systematic reviews and network meta-analyses have placed dextrose among the more promising injectates for symptom and functional improvement. These data cannot be directly extrapolated to post-surgical disease, but they support evaluating this intervention in a more difficult population rather than treating postoperative symptoms as an automatic indication for revision surgery [6,8,9].

Combining clinical, ultrasonographic, and electrodiagnostic assessment is particularly relevant here: in post-surgical CTS, symptoms alone are often insufficient to characterize the nature of ongoing pathology. Reviews of failed decompression emphasize the need for structured reassessment, while imaging and nerve conduction testing may clarify whether symptoms reflect persistent compression, incomplete release, scar-related tethering, or broader neuropathic dysfunction [3,37]. The parallel improvement in clinical outcomes and CSA indicates that the observed clinical benefit was accompanied by measurable structural change rather than relying on symptom reporting alone. Although recurrent CTS showed more favorable responder outcomes than persistent CTS, mixed-effects analyses of the continuous outcomes did not demonstrate statistically significant differences in longitudinal trajectories between subgroups.

Greater baseline median nerve enlargement was associated with a lower likelihood of treatment success, while recurrent rather than persistent postoperative CTS was associated with a more favorable response profile. This association should not be interpreted as indicating that CSA is a direct linear measure of clinical or electrodiagnostic severity. Rather, in this post-surgical cohort, greater nerve enlargement may have reflected a higher structural burden, including chronic swelling, fibrosis, impaired nerve gliding, or less reversible nerve change. A technical contribution is also possible, as larger or more adherent nerves may be more difficult to separate circumferentially, but the present study was not designed to quantify injectate spread or completeness of hydrodissection as an independent predictor. Severe electrodiagnostic abnormality showed an unfavorable pattern in the univariable analysis but did not remain in the final parsimonious model. Electrodiagnostic parameters showed modest improvement over follow-up, but these changes were smaller than the corresponding clinical and ultrasonographic improvements, consistent with the known lag of neurophysiologic recovery in chronic compressive neuropathy [27,38].

Patients with recurrent CTS needed fewer injections to achieve improvement, suggesting that when postoperative symptoms are driven primarily by scar formation and nerve tethering, the response to hydrodissection comes sooner and requires less retreatment. Persistent CTS, by contrast, showed lower success rates and a more frequent need for repeated treatment. These findings support a cautious, selective approach in persistent CTS, in which a limited response should prompt renewed diagnostic reassessment. The 12-month follow-up data further support the durability of the response. Among patients who met treatment success criteria at 6 months, 87.9% maintained their response at 12 months, with no significant difference between persistent and recurrent CTS (82.6% vs. 91.4%, *p* = 0.313). This finding suggests that when hydrodissection produces a meaningful early response, that response tends to be sustained rather than transient.

Collectively, these results point toward a selective role for D5W hydrodissection in post-surgical CTS, particularly in patients with recurrent symptoms after an initial period of satisfactory postoperative improvement and in those without marked baseline nerve enlargement. In persistent CTS, the lower response rate supports a more cautious interpretation and a lower threshold for renewed diagnostic reassessment.

Adverse events were minor or moderate, transient, and manageable, and no serious treatment-related complications were recorded. In a post-surgical population in whom revision surgery is not always immediately indicated, this safety profile supports consideration of hydrodissection as a low-risk non-surgical option warranting further controlled evaluation [20,39]. At a procedural level, these findings also support the value of anatomy-based ultrasound-guided interventions performed within a standardized institutional workflow, in which systematic scanning, continuous visualization of relevant neurovascular structures, and reproducible needle guidance are central to procedural safety and consistency [40,41]. Transient paresthesia was interpreted as a procedure-related mechanical phenomenon rather than an anesthetic-related effect, since no local anesthetic was injected into the perineural space. It may have reflected temporary pressure change within the carpal tunnel, tissue displacement during fluid separation, or transient irritation near the median nerve. All episodes resolved without persistent neurological deficit, and no nerve injury was observed.

This study addresses a population that remains underrepresented in the interventional literature, and it does so while maintaining the distinction between persistent and recurrent disease, a distinction that proved clinically meaningful. The multimodal evaluation approach reflects the diagnostic complexity of the post-surgical setting more closely than symptom reporting alone, and the composite endpoint, by requiring concurrent improvement across multiple domains, reduces the risk of overestimating benefit from a single favorable outcome measure.

This study has several limitations. First, the absence of a control group precludes definitive causal inference, and improvement cannot be attributed exclusively to hydrodissection or to D5W as injectate. Natural recovery after carpal tunnel release, regression to the mean, and spontaneous changes in nerve morphology or neurophysiology over time may have contributed to the observed findings. Second, the single-center design and performance of all procedures by one experienced operator may limit generalizability. Third, the persistent and recurrent subgroups differed in baseline clinical burden, so between-group comparisons should be interpreted cautiously despite adjusted analyses. Fourth, clinical assessors were not blinded to subgroup status, which may have influenced between-group outcome assessment. However, the composite primary endpoint relied predominantly on patient-reported components, including PGIC, pain intensity, and BCTQ scores, and the study also incorporated ultrasonographic and electrodiagnostic outcomes that were less directly dependent on evaluator judgment, although not fully immune to operator-related variability. Fifth, residual confounding related to rehabilitation exposure, splinting, analgesic escalation, and occupational wrist loading cannot be excluded. Systematic screening for hereditary neuropathy with liability to pressure palsies or amyloidosis was not performed in all participants; these conditions were excluded when previously known or clinically/electrodiagnostically suspected. Finally, classification as persistent or recurrent CTS depended partly on patient-reported postoperative symptom chronology, and the pragmatic six-month symptom-free interval used to define recurrence has not been externally validated.

The present study does not establish comparative efficacy against revision surgery or other interventional approaches, and it should not reduce the importance of careful reassessment in persistent postoperative symptoms. It does provide prospective, multimodal, subgroup-differentiated data in a population where such data have been lacking. Further controlled multicenter studies are needed to confirm these findings and refine patient selection.

## 5. Conclusions

In this prospective single-center cohort, ultrasound-guided 5% dextrose hydrodissection was associated with meaningful clinical and ultrasonographic improvement in selected patients with persistent or recurrent post-surgical carpal tunnel syndrome, with more favorable outcomes in recurrent disease. Baseline nerve enlargement and electrodiagnostic severity appeared to influence the likelihood of response. These findings offer prospective evidence in a population where such data have been scarce, with subgroup data that distinguish between persistent and recurrent disease and support further controlled multicenter studies to confirm efficacy and refine patient selection.

## Figures and Tables

**Table 1:** Baseline characteristics of the study population overall and by subgroup. Baseline demographic, clinical, ultrasonographic, and electrodiagnostic characteristics of the full cohort and according to post-surgical carpal tunnel syndrome subtype (persistent vs. recurrent).

Characteristic	Overall	Persistent CTS	Recurrent CTS	*p*-Value
Age, years	56.2 ± 11.8	58.6 ± 12.1	53.8 ± 11.0	0.041
Female sex, *n* (%)	71 (71.0)	37 (74.0)	34 (68.0)	0.66
Body mass index, kg/m^2^	29.0 ± 4.2	29.3 ± 4.4	28.7 ± 4.0	0.48
Diabetes mellitus, *n* (%)	28 (28.0)	18 (36.0)	10 (20.0)	0.12
Manual occupation, *n* (%)	57 (57.0)	30 (60.0)	27 (54.0)	0.69
Open surgery, *n* (%)	72 (72.0)	39 (78.0)	33 (66.0)	0.27
Endoscopic surgery, *n* (%)	28 (28.0)	11 (22.0)	17 (34.0)	0.27
Time since surgery, months	20.5 (11.0–32.5)	23.5 (13.2–43.8)	19.5 (10.0–29.2)	0.149
Duration of current symptoms, months	10 (5–20)	10.5 (7.0–23.0)	8.5 (4.0–16.0)	0.053
Baseline VAS	7.1 ± 1.3	7.4 ± 1.2	6.8 ± 1.3	0.018
Baseline BCTQ-SSS	3.66 ± 0.62	3.74 ± 0.63	3.58 ± 0.61	0.200
Baseline BCTQ-FSS	3.12 ± 0.57	3.21 ± 0.56	3.04 ± 0.58	0.139
CSA at inlet, mm^2^	14.7 ± 2.7	15.0 ± 2.8	14.5 ± 2.6	0.357
Electrodiagnostic severity, *n* (%)				0.29
Mild	20 (20.0)	8 (16.0)	12 (24.0)	
Moderate	48 (48.0)	22 (44.0)	26 (52.0)	
Severe	32 (32.0)	20 (40.0)	12 (24.0)	

Data are presented as mean ± SD, median (IQR), or *n* (%), as appropriate. Abbreviations: BCTQ-FSS, Boston Carpal Tunnel Questionnaire Functional Status Scale; BCTQ-SSS, Boston Carpal Tunnel Questionnaire Symptom Severity Scale; CSA, cross-sectional area; CTS, carpal tunnel syndrome; VAS, visual analogue scale.

**Table 2:** Primary and secondary outcomes at 6 and 12 months, including treatment exposure.

Outcome	Overall	Persistent CTS	Recurrent CTS	Absolute Difference (95% CI)	*p*-Value
At 6 months					
Treatment success, *n* (%) ^a^	58/100 (58.0)	23/50 (46.0)	35/50 (70.0)	24.0 (5.2 to 42.8)	0.015
PGIC indicating marked improvement, *n* (%) ^b^	60/94 (63.8)	23/46 (50.0)	37/48 (77.1)	27.1 (8.4 to 45.8)	0.006
CSA reduction ≥ 2 mm^2^, *n* (%) ^b^	53/94 (56.4)	20/46 (43.5)	33/48 (68.8)	25.3 (5.9 to 44.7)	0.014
Improvement by ≥1 Bland category, *n* (%) ^b^	27/94 (28.7)	11/46 (23.9)	16/48 (33.3)	9.4 (−8.7 to 27.6)	0.313
Revision surgery by 6 months, *n* (%)	3/100 (3.0)	2/50 (4.0)	1/50 (2.0)	−2.0 (−8.7 to 4.7)	0.558
At 12 months					
Treatment success, *n* (%) ^a^	52/100 (52.0)	20/50 (40.0)	32/50 (64.0)	24.0 (5.0 to 43.0)	0.016
PGIC indicating marked improvement, *n* (%) ^b^	54/91 (59.3)	21/45 (46.7)	33/46 (71.7)	25.1 (5.5 to 44.6)	0.015
CSA reduction ≥ 2 mm^2^, *n* (%) ^b^	48/91 (52.7)	18/45 (40.0)	30/46 (65.2)	25.2 (5.4 to 45.1)	0.016
Improvement by ≥1 Bland category, *n* (%) ^b^	35/91 (38.5)	14/45 (31.1)	21/46 (45.7)	14.5 (−5.2 to 34.3)	0.154
Maintained treatment success among 6-month responders, *n* (%) ^c^	51/58 (87.9)	19/23 (82.6)	32/35 (91.4)	8.8 (−9.2 to 26.9)	0.313
Cumulative revision surgery by 12 months, *n* (%)	6/100 (6.0)	4/50 (8.0)	2/50 (4.0)	−4.0 (−13.3 to 5.3)	0.400
Treatment exposure					
Median number of injections (IQR)	2 (1–3)	3 (2–3)	2 (1–2)	-	<0.001
1 injection, *n* (%)	31 (31.0)	9 (18.0)	22 (44.0)	-	-
2 injections, *n* (%)	29 (29.0)	13 (26.0)	16 (32.0)	-	-
3 injections, *n* (%)	24 (24.0)	16 (32.0)	8 (16.0)	-	-
4 injections, *n* (%)	16 (16.0)	12 (24.0)	4 (8.0)	-	-

^a^ Treatment success in the full analysis set; patients with missing 6- or 12-month primary endpoint data and those undergoing revision surgery before the corresponding assessment were classified as non-successes. ^b^ Calculated among patients with an observed assessment at the corresponding time point (6 months: *n* = 94; 12 months: *n* = 91). ^c^ Among patients meeting treatment success criteria at 6 months. Abbreviations: CSA, cross-sectional area; CTS, carpal tunnel syndrome; IQR, interquartile range; PGIC, Patient Global Impression of Change.

**Table 3:** Longitudinal changes in clinical and ultrasonographic parameters. Values are presented as observed mean ± SD over follow-up. *p*-values for time and time-by-group interaction were derived from linear mixed-effects models.

Parameter	Group	Baseline	3 Months	6 Months	12 Months	*p* for TIME	*p* for Time × Group
VAS	Overall	7.1 ± 1.3	4.1 ± 2.1	3.7 ± 1.4	3.8 ± 1.3	<0.001	0.172
	Persistent CTS	7.4 ± 1.2	4.8 ± 2.0	4.1 ± 1.3	4.1 ± 1.4	<0.001	
	Recurrent CTS	6.8 ± 1.3	3.4 ± 2.0	3.3 ± 1.5	3.4 ± 1.1	<0.001	
BCTQ-SSS	Overall	3.66 ± 0.62	2.76 ± 0.75	2.66 ± 0.75	2.66 ± 0.82	<0.001	0.626
	Persistent CTS	3.74 ± 0.63	2.95 ± 0.77	2.81 ± 0.72	2.85 ± 0.83	<0.001	
	Recurrent CTS	3.58 ± 0.61	2.58 ± 0.70	2.52 ± 0.75	2.49 ± 0.77	<0.001	
BCTQ-FSS	Overall	3.12 ± 0.57	2.42 ± 0.66	2.42 ± 0.68	2.45 ± 0.70	<0.001	0.546
	Persistent CTS	3.21 ± 0.56	2.58 ± 0.67	2.58 ± 0.63	2.60 ± 0.59	<0.001	
	Recurrent CTS	3.04 ± 0.58	2.26 ± 0.62	2.27 ± 0.69	2.31 ± 0.77	<0.001	
CSA at inlet, mm^2^	Overall	14.7 ± 2.7	13.5 ± 2.6	12.6 ± 3.1	12.7 ± 3.2	<0.001	0.551
	Persistent CTS	15.0 ± 2.8	14.1 ± 2.7	13.2 ± 3.4	13.4 ± 3.3	<0.001	
	Recurrent CTS	14.5 ± 2.6	12.9 ± 2.4	12.0 ± 2.8	12.1 ± 3.0	<0.001	

Abbreviations: BCTQ-FSS, Boston Carpal Tunnel Questionnaire Functional Status Scale; BCTQ-SSS, Boston Carpal Tunnel Questionnaire Symptom Severity Scale; CSA, cross-sectional area; CTS, carpal tunnel syndrome; VAS, visual analogue scale.

**Table 4:** Univariable associations with 6-month treatment success. Univariable logistic regression analyses of baseline variables associated with treatment success at 6 months.

Predictor	Crude OR	95% CI	*p*-Value
Recurrent vs. persistent CTS	2.74	1.20–6.23	0.016
Moderate vs. mild electrodiagnostic severity	0.71	0.23–2.19	0.556
Severe vs. mild electrodiagnostic severity	0.33	0.10–1.09	0.069
Baseline CSA, per 1 mm^2^ increase	0.84	0.71–0.98	0.026
Time since surgery, per 6 months	0.86	0.78–0.96	0.006
Diabetes mellitus	0.78	0.32–1.88	0.576
Age, per 10 years	0.84	0.60–1.19	0.324
Endoscopic vs. open surgery	0.52	0.22–1.26	0.147
Baseline BCTQ-SSS, per 1-point increase	0.85	0.45–1.62	0.623
Baseline VAS, per 1 point increase	0.97	0.71–1.32	0.841
Duration of current symptoms, per 6 months	1.00	0.98–1.01	0.713

Outcome: treatment success at 6 months in the full analysis set. Abbreviations: BCTQ-SSS, Boston Carpal Tunnel Questionnaire Symptom Severity Scale; CI, confidence interval; CSA, cross-sectional area; CTS, carpal tunnel syndrome; OR, odds ratio; VAS, visual analogue scale.

**Table 5:** Final multivariable model for 6-month treatment success. Parsimonious multivariable logistic regression model including recurrent versus persistent CTS status and baseline median nerve cross-sectional area.

Predictor	Adjusted OR	95% CI	*p*-Value
Recurrent vs. persistent CTS	2.63	1.14–6.10	0.024
Baseline CSA, per 1 mm^2^ increase	0.84	0.72–0.99	0.038

Model performance: apparent C-statistic = 0.68; optimism-corrected C-statistic after bootstrap validation = 0.66; calibration slope = 1.00; Hosmer–Lemeshow *p* = 0.182; all VIF values ≤ 1.01. Abbreviations: CI, confidence interval; CSA, cross-sectional area; CTS, carpal tunnel syndrome; OR, odds ratio; VIF, variance inflation factor.

**Table 6:** Adverse events and revision surgery. Safety outcomes in the overall cohort and by post-surgical carpal tunnel syndrome subtype.

Event	Overall (*n* = 100)	Persistent CTS (*n* = 50)	Recurrent CTS (*n* = 50)
Any adverse event, *n* (%)	22 (22.0)	13 (26.0)	9 (18.0)
Injection-site pain > 24 h, *n* (%)	11 (11.0)	6 (12.0)	5 (10.0)
Bruising/ecchymosis, *n* (%)	5 (5.0)	3 (6.0)	2 (4.0)
Transient paresthesia, *n* (%)	7 (7.0)	4 (8.0)	3 (6.0)
Hematoma requiring observation, *n* (%)	2 (2.0)	1 (2.0)	1 (2.0)
Prolonged pain > 48 h, *n* (%)	1 (1.0)	1 (2.0)	0 (0.0)
Infection, *n* (%)	0	0	0
Nerve injury, *n* (%)	0	0	0
Tendon injury, *n* (%)	0	0	0
Serious adverse events, *n* (%)	0	0	0
Revision surgery by 6 months, *n* (%)	3 (3.0)	2 (4.0)	1 (2.0)
Cumulative revision surgery by 12 months, *n* (%)	6 (6.0)	4 (8.0)	2 (4.0)

No serious treatment-related adverse events were observed. Abbreviations: CTS, carpal tunnel syndrome.

## Data Availability

The original contributions presented in this study are included in the article. Further inquiries can be directed to the corresponding authors.

## Supplementary Materials

The following supporting information can be downloaded at: https://www.mdpi.com/article/10.3390/diagnostics16132106/s1, Video S1: Ultrasound-guided 5% dextrose hydrodissection of the median nerve in post-surgical carpal tunnel syndrome; Table S1: Electrodiagnostic parameters over follow-up in the overall cohort and by subgroup.

## Abbreviations

The following abbreviations are used in this manuscript:

BCTQ-FSS	Boston Carpal Tunnel Questionnaire Functional Status Scale
BCTQ-SSS	Boston Carpal Tunnel Questionnaire Symptom Severity Scale
CI	confidence interval
CMAP	compound muscle action potential
CSA	cross-sectional area
CTS	carpal tunnel syndrome
D5W	5% dextrose in water
IQR	interquartile range
OR	odds ratio
PGIC	Patient Global Impression of Change
SD	standard deviation
SNAP	sensory nerve action potential
SPSS	Statistical Package for the Social Sciences
STROBE	Strengthening the Reporting of Observational Studies in Epidemiology
VAS	visual analogue scale
VIF	variance inflation factor
TCL	transverse carpal ligament
Sca	scaphoid
FPL	flexor pollicis longus
MN	median nerve
Pis	pisiform
Cap	capitate
FDS2	flexor digitorum superficialis digit 2
FDS3	flexor digitorum superficialis digit 3
FDS4	flexor digitorum superficialis digit 4
FDS5	flexor digitorum superficialis digit 5
FDP2	flexor digitorum profundus digit 2
FDP3	flexor digitorum profundus digit 3
FDP4	flexor digitorum profundus digit 4
FDP5	flexor digitorum profundus digit 5
