# Supplementary material for: Ultrasound-Guided 5% Dextrose Hydrodissection Procedures for Persistent and Recurrent Post-Surgical Carpal Tunnel Syndrome: A Prospective Single-Center Cohort Study
Source: Diagnostics (Basel). 2026 Jul 5;16(13):2106. doi: 10.3390/diagnostics16132106 (PMC13361386; doi:10.3390/diagnostics16132106)
Supplement: Supplementary file 1 [file diagnostics-16-02106-s001.zip › diagnostics-4425509-supplementary.pdf]

Parameter	Group	Baseline (n=100)	6 months (n=94)	12 months (n=91)
Median sensory latency, ms	Overall	4.56 ± 0.77	4.19 ± 0.81	4.10 ± 0.82
	Persistent CTS	4.69 ± 0.80	4.41 ± 0.84	4.31 ± 0.86
	Recurrent CTS	4.43 ± 0.72	3.97 ± 0.72	3.89 ± 0.74
Median distal motor latency, ms	Overall	5.20 ± 0.91	4.81 ± 0.86	4.74 ± 0.88
	Persistent CTS	5.38 ± 0.96	5.06 ± 0.92	4.98 ± 0.94
	Recurrent CTS	5.02 ± 0.83	4.58 ± 0.73	4.50 ± 0.75
SNAP amplitude, μV	Overall	12.2 ± 5.4	13.9 ± 5.7	14.6 ± 5.9
	Persistent CTS	11.4 ± 5.0	12.6 ± 5.2	13.1 ± 5.3
	Recurrent CTS	13.0 ± 5.7	15.2 ± 5.9	16.1 ± 6.1
CMAP amplitude, mV	Overall	6.9 ± 2.0	7.3 ± 2.0	7.5 ± 2.1
	Persistent CTS	6.6 ± 1.9	6.9 ± 1.9	7.1 ± 2.0
	Recurrent CTS	7.2 ± 2.1	7.7 ± 2.1	7.9 ± 2.2

Table S1. Electrodiagnostic parameters over follow-up in the overall cohort and by subgroup. SNAP - sensory nerve action potential; CMAP - compound muscle action potential
